# Cardiac Hemodynamics, Tissue Oxygenation, and Functional Capacity in Post-COVID-19 Patients

**DOI:** 10.3390/medicina61010124

**Published:** 2025-01-14

**Authors:** Elizane Poquiviqui do Nascimento, Larissa Fernanda Estevam do Nascimento, Lhara de Freitas Castro, Vilena Cavalcante de Barros, Emily Rachel Pereira Bandeira, Thiago Bezerra Wanderley e Lima, Matías Otto-Yáñez, Guilherme Augusto de Freitas Fregonezi, Vanessa Regiane Resqueti

**Affiliations:** 1 Laboratório de Inovação Tecnológica em Reabilitação, Departamento de Fisioterapia, Universidade Federal do Rio Grande do Norte (UFRN), Campus Universitário Central, Natal 59078970, RN, Brazil; elipoquiviqui@gmail.com (E.P.d.N.); larissa.nascimento.081@ufrn.edu.br (L.F.E.d.N.); lhara.castro.099@ufrn.edu.br (L.d.F.C.); vilena.cavalcante@gmail.com (V.C.d.B.); emilly.bandeira.701@ufrn.edu.br (E.R.P.B.); thiagowanderley13@hotmail.com (T.B.W.e.L.); guilherme.fregonezi@ufrn.br (G.A.d.F.F.); 2 PneumoCardioVascular Lab/HUOL, Hospital Universitário Onofre Lopes, Universidade Federal do Rio Grande do Norte (UFRN), Natal 59012300, RN, Brazil; 3 Grupo de Investigación en Salud, Funcionalidad y Actividad Física (GISFAF), Facultad de Ciencias de la Salud, Universidad Autónoma de Chile, Santiago 8320000, Chile; matias.otto@uautonoma.cl

**Keywords:** exercise tolerance, exercise test, coronavirus infections, cardiograph, impedance, hemodynamics, spectroscopy, near infrared

## Abstract

*Background and Objectives*: This study aimed to evaluate and compare the functional capacity of post-COVID-19 patients with a control group and analyze cardiac hemodynamics and muscle tissue oxygenation responses during assessment protocols in both groups. *Materials and Methods*: A cross-sectional study was conducted involving patients with COVID-19 and a control group who were all aged ≥18 years. Participants underwent two functional capacity tests: the one-minute sit–stand test (1-STS) and the six-minute walk test (6MWT). Cardiac hemodynamic responses were evaluated using impedance during the 1-STS, and tissue perfusion responses in the oxygenation were recorded during and after both tests. The Friedman test was used for within-group and the Mann–Whitney test was used for between-group comparisons. *Results*: Thirty-six post-COVID-19 patients (median age 36 years, BMI 26.51 kg/m^2^) and eleven control subjects (median age 25 years, BMI 23.71 kg/m^2^) were enrolled. The post-COVID-19 group showed a 20% decrease in 6MWT distance (*p* = 0.0001) and a 28% decrease in 1-STS repetitions (*p* = 0.01) versus the control group. Cardiac hemodynamic differences were observed in the post-COVID-19 group during the 1-STS, with reductions in the stroke volume index (18%, *p* = 0.004), cardiac index (21%, *p* = 0.0009), Contractility Index (78%, *p* = 0.0001), and Ejection Fraction (29%, *p* = 0.0003) and increases in Systemic Vascular Resistance (25%, *p* = 0.03) and the Systemic Vascular Resistance Index (27%, *p* = 0.0007). Tissue oxygenation during the 6MWT and 1-STS showed no significant differences between groups. *Conclusions*: The post-COVID-19 subjects exhibited a reduction in functional capacity, changes in hemodynamic responses related to cardiac and systemic vascular resistance, and a similar pattern of muscle oxygen delivery and consumption in both tests

## 1. Introduction

Coronavirus disease 2019 (COVID-19) is known to be associated with a wide range of disorders varying in type and severity and affecting the respiratory, physical, and psychological aspects of patients [1]. In severe cases, patients often experience prolonged immobility as they require intensive care for critical illness. This immobility can lead to a variety of complications, including cardiorespiratory issues, physical deconditioning, postural instability, venous thromboembolism, muscle shortening, contractures (myogenic, neurogenic, atherogenic), and injuries resulting from pressure and acquired weakness in the intensive care unit (ICU) [2].

ICU-acquired weakness is strongly associated with a variety of adverse outcomes, including prolonged reliance on mechanical ventilation (MV), extended ICU and hospital stays, increased morbidity and mortality, increased healthcare costs, and various functional limitations [3]. In addition, this condition can have a persistent negative impact on quality of life for up to five years after hospitalization [4]. These consequences have significant socioeconomic implications, with nearly one-third of patients with ICU-acquired weakness unable to return to work and another third unable to return to their previous role or maintain a similar salary level [5]. Therefore, given the severity of COVID-19 and its association with prolonged immobility in severely affected patients, understanding and proactively addressing these outcomes is fundamental [6].

Recent studies provide additional evidence of the medical implications of COVID-19, requiring medical attention for both short- and long-term manifestations that persist for a period of three months to one year after the initial illness [7,8,9].

Specifically, COVID-19 infection can result in both short- and long-term respiratory or systemic complications [10], including documented cardiovascular complication issues such as myocarditis, myocardial infarction, heart failure, cardiogenic shock, and arrhythmias [11]. Additionally, there appears to be an association between COVID-19 and persistent cardiac injury even after recovery, particularly in the form of short-term subclinical myocardial injury and long-term diastolic dysfunction [12].

As a respiratory consequence, diffuse alveolar damage may occur, resulting in decreased systemic and tissue oxygenation [13]. Consequently, individuals affected by COVID-19 may experience impaired oxygenation of their musculoskeletal system during exercise due to decreased oxygen delivery resulting from decreased cardiac output and/or arterial oxygen concentration. This deficiency in peripheral muscle microcirculation and reduced skeletal muscle capillary density could affect exercise tolerance and quadriceps strength similar to the effects observed in patients with chronic obstructive pulmonary disease (COPD) who experience reduced oxygen delivery to active muscles during dynamic exercise [14,15]. In other conditions characterized by changes in pulmonary gas exchange, reduced delivery of oxygen to active muscles during dynamic exercise has been observed, such as in patients with COPD [16]. However, the specific impact of such changes during exercise in post-COVID-19 patients remains unclear.

Functional capacity in post-hospitalized patients can be assessed using a variety of tools. Currently, the 6 min walk test (6MWT) and the 1 min sit-to-stand test (1-STS) are the most commonly used functional tests to assess functional capacity in post-COVID-19 patients [17]. These tests have been shown to be effective in identifying impaired functional capacity, as evidenced by decreased exercise capacity and oxygen saturation during exertion after hospital discharge for this condition [18,19,20].

This study hypothesizes that post-COVID-19 patients experience a reduction in functional capacity, possibly associated with changes in peripheral muscles due to discrepancies and deficiencies in cardiac function and peripheral tissue oxygenation. Therefore, the main objective of this study is to analyze and compare exercise capacity, cardiac hemodynamics, and tissue oxygenation during functional capacity testing in post-COVID-19 adults.

## 2. Materials and Methods

### 2.1. Study Design and Participants

This cross-sectional study was conducted between July 2020 and March 2022 and included 47 volunteers of both genders over 18 years with no history of cardiovascular, restrictive, and/or obstructive pulmonary disease. Exclusion criteria were individuals with neurological, cognitive, or osteoarticular impairments that could affect data collection or those who did not complete the evaluation. The COVID-19 group consisted of 36 subjects who tested positive for COVID-19 (hospitalized or not) and were confirmed by RT-PCR. The control group consisted of 11 self-reported healthy individuals, matched by age and body mass index (BMI) to the COVID-19 group, without respiratory, cardiac, and/or neuromuscular disease and normal spirometric values. In this study, neither the COVID-19 group nor the control group received any COVID-19 vaccinations prior to the study period. While we cannot guarantee that the control group was not infected with COVID-19, we can confirm that they did not exhibit any symptoms or manifestations of the virus. This study adhered to the Declaration of Helsinki and was approved by the Research Ethics Committee of the Hospital Universitário Onofre Lopes (HUOL/EBSERH) under protocol number 4.172.356.

### 2.2. Study Protocol

All subjects received detailed information about the study procedures and signed an informed consent form. The evaluation protocol was performed in the following order: medical history, pulmonary function testing, 1-STS with tissue perfusion assessment and electrical impedance hemodynamics, and 6MWT with tissue perfusion assessment.

### 2.3. Pulmonary Function

Pulmonary function was assessed using the Master Screen Diffusion RT spirometer (CareFusion, San Diego, CA, USA) according to the American Thoracic Society/European Respiratory Society (ATS) reproducibility and acceptability criteria [21]. Absolute and percentage values of predicted forced vital capacity (FVC), forced expiratory volume in one second (FEV1), and FEV1/FVC ratio were considered and compared with reference values for the Brazilian population [22].

### 2.4. One-Minute Sit-to-Stand Test (1-STS)

The 1-STS was performed according to the protocol by Ozalevli et al. [23]. Participants were instructed to place their arms around their chest and perform repeated sit-to-stand movements as quickly as possible within the 1 min time frame. A standard 46 cm high with back support and no armrests was used for the test. Heart rate (HR), peripheral oxygen saturation (SpO_2_ (Nonin 2500A, Plymouth, MN, USA), blood pressure (BP) (Omron HEM-7320-BR), and symptoms of dyspnea and fatigue (assessed using the Borg 0–10 scale) were measured before the test, immediately after its completion, and two minutes after the test. To ensure accuracy, two repetitions of the 1-STS test were performed with a 30 min interval between them. Reference values for comparison were obtained from the study conducted by Strassmann et al. [24].

### 2.5. Six-Minute Walk Test (6MWT)

The 6MWT was used to assess the clinical functional exercise capacity according to the ATS recommendations [25]. Participants walked for six minutes in a 30 m, flat, covered corridor, with each meter marked on the ground with a tape measure and the return marked by a cone at each end. During the test, we recorded heart rate (HR), peripheral oxygen saturation (SpO_2_), blood pressure (BP), and dyspnea using the modified Borg scale (0–10). Standardized encouragement phrases were provided every minute in a consistent tone of voice. At the end of the test, the total distance walked was calculated, and all previously assessed parameters were immediately reassessed. The predicted walking distance for this test was calculated according to the method proposed by Iwama et al. [26].

### 2.6. Electrical Impedance Hemodynamics

Impedance cardiography was performed during 1-STS using Physio Flow (Manatec, Paris, France). Hemodynamic parameters were measured continuously during preparation for the 1-STS at rest, during the test, and two minutes after completion (recovery). Parameters measured included stroke volume (SV, mL), the stroke volume index (SVi, mL/m^2^), heart rate (HR, bpm), cardiac output (CO, L/min), the cardiac index (CI, mL/min/m^2^), Systemic Vascular Resistance (SVR, Din.s/cm^5^), the Systemic Vascular Resistance Index (SVRi, Din.s/cm^5^.m^2^), Ejection Fraction (EF, %), End Diastolic Volume (EDV, mL), Ventricular Ejection Time (VET, ms), and the Contractility Index (CTI). SV, CO, and SVR values were indexed to body surface area to calculate Svi, CI, and SVRi, respectively. The application areas were previously prepared by shaving and cleaning the skin with alcohol followed by abrasion with Nuprep gel (Weaver and Company, Aurora, CO, USA). Electrodes were placed above the supraclavicular fossa at the left base of the neck and along the spinal line at the thoracolumbar junction.

### 2.7. Tissue Perfusion Assessment

Oxyhemoglobin (O2Hb), deoxyhemoglobin (HHb), total hemoglobin (tHb), and the tissue saturation index in muscle tissue (TSI) were measured using wireless Near-Infrared Spectroscopy (NIRS) with the Portmon device (Artinis, Elst, The Netherlands) during and up two minutes after the two functional tests. Data recorded by the TX3 LED emitter, which captures signals from deeper tissues, were used for analysis to minimize potential measurement bias due to adipose tissue thickness and cutaneous blood flow. Data analysis was performed using proprietary Oxysoft software (Oxysoft, Artinis Medical Systems, BV, Elst, The Netherlands).

The NIRS device was carefully attached to the belly of the vastus lateralis muscle of the dominant lower limb using double-sided adhesive tape, and an elastic bandage was wrapped around it to ensure stability and prevent disturbances during the tests. This standardized and precise application of the NIRS device allowed for accurate measurements of hemoglobin levels and tissue oxygen saturation during the functional tests, facilitating a comprehensive analysis of muscle oxygenation responses in post-COVID-19 subjects and the control group.

### 2.8. Statistical Analysis

To determine the sample size for this study, a pilot study was conducted with 8 subjects who had previously contracted COVID-19 (3 male and 5 female) and 4 controls, who were matched for sex and age. The effect size (r) was estimated to be 0.65, with an alpha error set at 0.05 and a statistical power of 80%. These parameters were used in the G*Power program version 3.1 (Heinrich-Heine-Universität Düsseldorf, Düsseldorf, Germany) to calculate the sample size based on the formula described in Fritz, Moris, and Richer, 2012. [27].

Data are expressed as median and interquartile range. Normality and distribution of data were assessed using the Shapiro–Wilk test. Intragroup comparisons of tissue perfusion and cardiac hemodynamic variables by impedance were performed using the Friedman test, and intergroup comparisons were performed using the Mann–Whitney test. Data were analyzed using the GraphPad Prism 8.0 statistical program (GraphPad Software Inc., San Diego, CA, USA) with a significance level of *p* < 0.05 and a 95% confidence interval (CI). The effect size and power of this study were determined using G*power 3.1 software (Heinrich-Heine-Universität Düsseldorf, Düsseldorf, Germany), with an effect size of 2.01 and statistical power of 0.99 based on the distance walked in the 6MWT as the main variable.

## 3. Results

Thirty-six individuals who had COVID-19 and eleven controls agreed to participate in the research. Table 1 shows the characteristics of the two groups. In the post-COVID-19 group, two people (5.5%) had systemic arterial hypertension (SAH), 2.7% had congestive heart failure (CHF) diagnosed after COVID-19, one person (2.7%) had diabetes mellitus (DM), four people (11.11%) were self-reported smokers, twenty-three (63.88%) were alcoholics, and one person (2.7%) was diagnosed with depression. In the control group, one person (9.09%) was a smoker, six (54.54%) were alcoholics, and one (9.09%) was diagnosed with depression. Only sixteen (44.5%) of the post-COVID-19 group engaged in regular physical activity compared to eight (72.72%) of the control group. The post-COVID-19 group showed a reduction in FEV1, FEF25–75%, and PEF compared to the control group, but the differences were not statistically significant.

In the post-COVID-19 group, 16.66% had undergone invasive mechanical ventilation (IMV) during their hospitalization. On average, they received IMV for 17.2 days and spent 22.33 days in the intensive care unit (ICU) and 32.33 days in the hospital. In the subgroup of individuals who received IMV, there were no statistically significant differences in spirometry values compared to the control group.

### 3.1. Functional Capacity

#### 3.1.1. 1-STS

The results of the 1-STS, cardiorespiratory parameters, and cardiac hemodynamics are summarized in Table 2. In the 1-STS assessment, we observed a significant reduction in functional capacity in the COVID-19 group, which was approximately 28% (*p* = 0.01). However, no desaturation was observed in any of the groups, and there was no statistical difference between the groups regarding reported symptoms of dyspnea and post-exercise fatigue. Patients requiring hospitalization and invasive mechanical ventilation (IMV) had a median of 35.5 (26–44) STS repetitions and 74.3% (51.61–86.23) of predicted values.

We observed a statistically significant intergroup difference in the assessment during the 1-STS in the post-COVID-19 group, with a reduction of 18% in SVi (*p* = 0.004), 21% in CI (*p* = 0.0009), 78% in CTI (*p* = 0.0001), and 29% in EF (*p* = 0.0003). In addition, there was a 25% increase in SVR (*p* = 0.03) and a 27% increase in SVRi (*p* = 0.0007).

Table 3 shows the hemodynamic data for both groups during the performance of the 1-STS at different time points: rest, start of the 1-STS, end of the test, and recovery (two minutes after completion). The value of *p* intragroup analysis presents the hemodynamics difference between the observed moments within each group. The *p*-value for intergroup analysis shows the difference between the groups at each moment.

As expected, we observed statistically significant intragroup differences. Both groups showed large responses to cardiac variables at the end of the functional test.

The main intergroup findings include significant differences in EF, EDV, and CTI at all analyzed moments. The CI and SVRi data showed differences at the start and end moments, while SVi and HR showed differences only at the start moment.

#### 3.1.2. 6MWT

Table 4 provides a detailed performance analysis of both groups and the variables measured by the 6MWT. The intergroup analysis showed a reduction of approximately 20% in performance in the COVID-19 group (*p* = 0.0001), which corresponds to a median reduction of 105 m. This reduction was approximately 18% of the predicted percentage when compared to the performance of the control group (*p* < 0.0001). Hospitalized patients using IMV achieved a median 6MWT distance of 485.5 (447.5–557.3) meters or 84.42% (74.44–89.83) of the predicted value.

### 3.2. Tissue Perfusion

**Figure 1:**
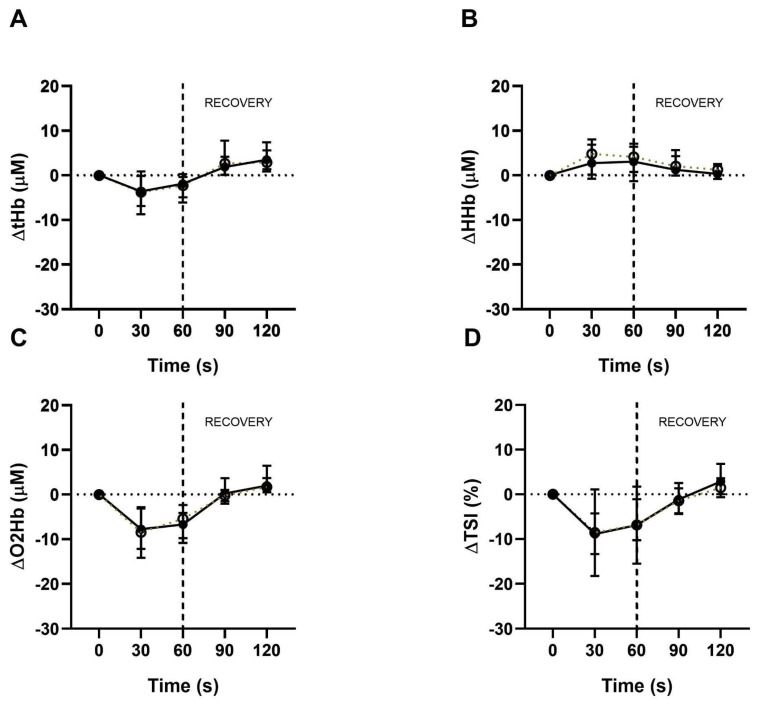
Tissue perfusion during the 1-STS and recovery. Representative data of (**A**) ΔtHb, (**B**) ΔHHb, (**C**) ΔO2Hb, and (**D**) ΔTSI obtained by NIRS every thirty seconds from the 1-STS and during recovery from COVID-19 (markers, closed markers, and full line) and control (open markers and dotted line); presented in median and interquartile range.

**Figure 2:**
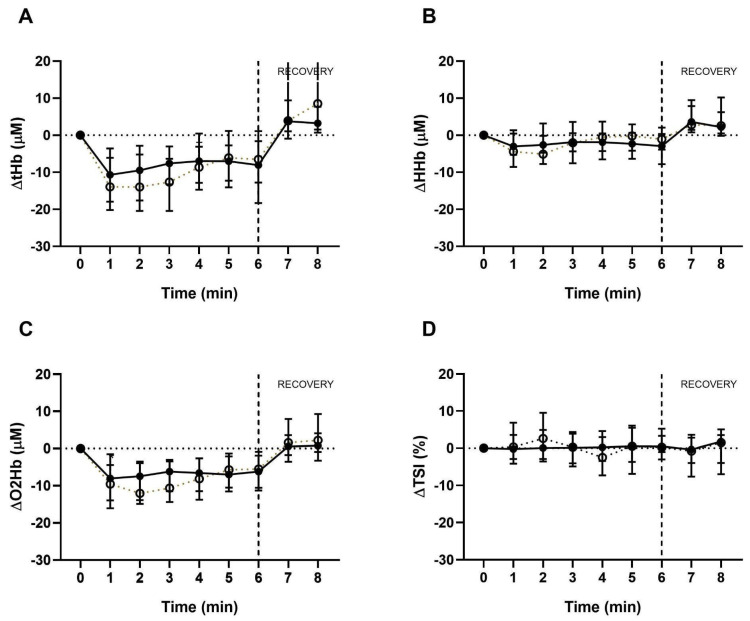
Tissue perfusion during the 6MWT and recovery. Representative data of (**A**) ΔtHb, (**B**) ΔHHb, (**C**) ΔO2Hb, and (**D**) ΔIST obtained by NIRS at each 6MWT minute and each COVID-19 recovery minute (markers, closed markers, and full line) and control (open markers and dotted line); presented in median and interquartile range.

The tissue oxygenation response in both functional capacity tests is shown in Figure 1 and Figure 2. Compared to the baseline, a pattern of decrease in ΔtHb, ΔO2Hb, and ΔTSI was observed at the beginning of both functional capacity tests. During the test, these curves tended to stabilize and showed a subsequent increase in these variables at recovery.

There was a slight increase in ΔHHb during the 1-STS (Figure 1B) and a subsequent decrease during recovery. In the 6MWT, this variable showed a similar pattern to the others (Figure 2). Changes in tissue perfusion curves were of similar magnitude between the COVID-19 group and the control group, with no statistically significant intergroup difference in the studied variables.

## 4. Discussion

The aim of this study was to investigate the influence of COVID-19 on the functional capacity of individuals who had recovered from infection. Our results suggest several important observations when comparing the post-COVID-19 group with uninfected individuals: (I) a reduction in functional capacity identified by both tests, by 28% in the 1-STS and 20% in the 6MWT, (II) changes in cardiac hemodynamic responses and systemic vascular resistance, and (III) a similar pattern of muscular oxygen delivery and consumption in the two functional tests in both groups.

To assess functional capacity, we used two functional tests that have been widely used in previous studies [28]. Our results are consistent with the findings of Eksombatchai et al. (2021) who evaluated the 6MWT performance of 87 COVID-19 patients with different levels of disease severity (mild flu symptoms (51%), severe pneumonia (8%), and mild pneumonia (40%) [29]. Although we did not find a statistically significant difference between the groups (*p* = 0.118), the group with severe pneumonia showed a reduction in the distance covered in the 6MWT, while the groups with mild flu symptoms and mild pneumonia had mean 6MWT distances of 538 ± 56.8 and 527.5 ± 53.5 m, respectively.

In the 1-STS, we observed a significant 28% reduction in the number of repetitions (*p* = 0.01), which is consistent with the results of other studies. Belli et al. (2020) [30] evaluated 103 patients after hospitalization for COVID-19 and found a reduced performance on the 1-STS equivalent to 74.4% (14 ± 6 repetitions) of the predicted value for them. Similarly, Paneroni et al. (2021) evaluated 41 patients after hospital discharge for COVID-19 and found a performance of 63% (22.1 ± 7.3 repetitions) on the 1-STS. It is important to note that our results show a smaller magnitude of reduction, likely due to the fact that our patients were not evaluated immediately after hospital discharge [30]. As described in the literature, SARS-Cov-2 infection affects the cardiovascular system in several ways. Myocardial injury is detected in 25% of hospitalized COVID-19 patients and is associated with a high risk of mortality [31]. The incidence of more serious cardiovascular events, such as acute myocardial infarction type I and II, associated with SARS-Cov-2 infection, significantly increases the risk of cardiac injury. Macroscopic histopathologic findings at necropsy of patients with COVID-19 have shown evidence of chronic heart disease, including myocardial hypertrophy (92.9%), mild to severe coronary artery atherosclerosis (100%), and focal myocardial fibrosis (21.4%), with acute myocardial infarction being a concomitant cause of death in 21.4% of patients [32]. In our study, we found a reduction of 18% in SVi (*p* = 0.004), 21% in CI (*p* = 0.0009), 78% in CTI (*p* = 0.0001), and 29% in EF (*p* =0.0003), along with an increase in SVR by 25% (*p* = 0.03) and 27% in SVRi (*p* = 0.0007) in post-COVID-19 patients, suggesting impaired cardiac function in these individuals. Furthermore, SVRi showed intergroup differences at baseline (*p* = 0.001) and at the end of functional testing (*p* = 0.001). The increase in peripheral vascular resistance is thought to be due to the high sympathetic outflow present in COVID-19, which can also significantly compromise coronary perfusion [33,34]. Sympathetic hyperactivity has been identified as an independent predictor of mortality in several diseases, including cardiovascular disease [35]. A review of microneurography studies of the sympathetic nervous system by Vallbo et al. (2004) showed a significant increase in sympathetic activity in patients with heart failure compared to controls, along with other relationships related to arterial baroreflex activity [36]. The exact molecular mechanisms underlying COVID-19-induced autonomic dysregulation are not fully understood, but it is known that SARS-CoV-2 infection is associated with high levels of inflammatory cytokines that can have significant effects on the respiratory, vascular, and nervous systems. These cytokines have the ability to cross the blood–brain barrier, thereby increasing sympathetic nervous system activation [37].

During exercise, changes in tissue O_2_ can be observed by a decrease in oxyhemoglobin (O2Hb) and an increase or constant level of deoxyhemoglobin (HHb) [38], as observed in both functional tests, indicating an increase in the oxygen demand of the muscle being tested. The sum of O2Hb and HHb (tHb) reflects the total amount of hemoglobin (Hb) and myoglobin (Mb) in the tested tissue. The total Mb concentration does not change acutely during exercise; therefore, changes in tHb reflect vasodilation or an increase in capillary hematocrit in the tissue under investigation [39]. Despite the decrease in tHb and O2Hb in both groups, no statistically significant difference in blood flow or O_2_ delivery and consumption was observed during the functional tests.

Similar results have been found in patients with chronic respiratory diseases, such as those with COPD. Layec et al. (2017) [40] studied 24 individuals, 12 with non-hypoxemic COPD and 12 healthy sedentary individuals, and subjected them to dynamic plantar flexion exercise at 40% of their maximal work rate with assessment by NIRS. Similar to our study, the groups showed no difference in O_2_ delivery during exercise and recovery. This suggests that O_2_ consumption and O_2_ delivery (O2Hb and HHB) appear to adequately match muscle metabolic demands in both healthy controls and chronically ill patients. It was also observed that metabolic recovery and mitochondrial capacity in patients were not significantly different from controls, indicating that metabolic recovery in the skeletal muscle of patients is preserved despite the hemodynamic changes.

An increase in the tissue saturation index (TSI) was observed in both groups during the recovery phase of both functional tests. This increase in the TSI, along with the increase in O2Hb, may be associated with increased skin blood flow, possibly for thermoregulatory purposes.

These findings suggest potential respiratory impairments and the influence of various comorbidities and lifestyle factors in the post-COVID-19 group. However, larger sample sizes and further investigations are needed to draw more definitive conclusions about the effect of COVID-19 on lung function and the association with mechanical ventilation and other variables.

### Limitations

The present study has certain limitations, including the heterogeneity of the COVID-19 sample and the relatively small sample size, which may affect the results. Nevertheless, our findings are highly relevant in the global context of the persistent changes observed in patients with ongoing COVID-19 during the pandemic. To extend and strengthen these findings, we recommend further investigation with larger sample sizes and stratification of post-COVID-19 groups. Such efforts would increase the comprehensiveness of the research and provide additional insight into the lasting effects of COVID-19 on affected individuals.

## 5. Conclusions

In conclusion, both functional tests detected performance differences between the post-COVID-19 group and the uninfected control group, indicating altered cardiac hemodynamics in individuals who had COVID-19 during functional activities. Although functional performance differed, post-COVID-19 patients had unchanged tissue oxygenation, including delivery and muscle flow during exercise. These findings emphasize the importance of assessing cardiac responses in post-COVID-19 individuals during functional tasks and contribute to our understanding of the virus’s lasting effects on cardiovascular function and exercise capacity.

## Figures and Tables

**Table 1:** Sample characteristics.

Variables	COVID-19 (n = 36)	CONTROL (n = 11)	*p*
Age (years)	36 (29–51)	25 (23–39)	0.007 *
Weight (kg)	76.95(67.73–89.3)	64.5 (57.7–73.7)	0.04 *
Height (cm)	167.5(162–175)	160 (159–167)	0.07
**Sex [n (%)]**			
F	13 (36.11)	8 (72.72)	-
M	23 (63.88)	3 (27.27)	-
BMI (kg/m^2^)	26.51 (24.06–30.32)	23.71 (22.54–28.28)	0.14
Length of stay (days)	13 (5–33)	-	-
Use of IMV (days)	0 (0–13)	-	-
**Comorbidities [n (%)]**			
SAH	2 (5.5)	0	-
CHF	1 (2.7)	0	-
DM	1 (2.7)	0	-
Smoking	4 (11.11)	1 (9.09)	-
Alcoholism	23 (63.88)	6 (54.54)	-
Depression	1 (2.7)	1 (9.09)	-
**Spirometry data**			
FVC (L)	3.82 (3.048–4.388)	3.88 (3.45–4.76)	0.65
FVC (%Pred)	90.02 (75.69–100.2)	99.14 (91.45–104.4)	0.22
FEV_1_ (L)	2.91 (2.55–3.48)	3.31 (2.98–3.97)	0.2
FEV_1_ (%Pred)	83.04 (75.09–98.74)	101.2 (79.22–115.2)	0.24
FEV_1_/FCV	82.55 (78.46–86.39)	85.13 (79.77–90.96)	0.39
FEF_25–75%_ (L/s)	3.38 (2.58–4.01)	3.86 (3.04–5.44)	0.39
FEF_25–75%_ (%Pre)	81.57 (67.45–107.2)	96.91 (62.06–131.2)	0.78
PEF (L/s)	4.72 (3.75–5.97)	7.00 (4.4–8.32)	0.14

Data presented in median and interquartile ranges and percentage. Captions: BMI: body mass index; IMV: invasive mechanical ventilation; F: female; M: male; SAH: systemic arterial hypertension; CHF: congestive heart failure; DM: diabetes mellitus; FVC: forced vital capacity; FEV_1_: forced expiratory volume in the first second; FEV_1_/FCV: FEV_1_/FCV ratio; FEF_25–75%_: forced mid-expiratory flow; PEF: Peak Expiratory Flow; * difference between groups *p* < 0.005.

**Table 2:** 1-STS results, cardiorespiratory parameters, and hemodynamics by impedance.

1-STS	COVID-19 (n = 36)	CONTROL (n = 11)	*p*
1-STS (repeats)	33.5 (27.5–41.75)	43 (35–51)	0.01 *
1-STS (% pred)	73.55 (64.88–86.82)	85.07 (65.44–115.4)	0.05
SpO_2_ post (%)	98 (97–98)	98 (97–98)	0.69
HR post (bpm)	107 (96.5–127.3)	115 (100–142)	0.25
Dyspnea post (Borg 0–10)	4 (2.25–5.75)	4 (3–5)	0.8
Fatigue post (Borg 0–10)	5 (3–7)	5 (4–6)	0.67
**Hemodynamics by impedance data**			
HR (bpm)	117.8 (105.9–131.2)	125.4 (111–141.2)	0.19
SV (mL)	86.2 (70.55–95.86)	87.20 (77.30–110.8)	0.46
SVi (mL/m^2^)	43.63 (39.12–50.02)	51.56 (45.8–53.2)	0.004 *
CO (L/min)	9.99 (8.242–11.57)	11.84 (8.664–13.32)	0.11
CI (mL/min/m^2^)	5.32 (4.545–6.081)	6.45 (6.1–7.023)	0.0009 *
CTI	196.6 (143.3–253.9)	350.1 (303.3–467.9)	0.0001 *
VET (ms)	235.2 (213.8–275.5)	254.8 (219.8–272)	0.66
SVR (Din.s/cm⁵)	714.5 (602.2–842.4)	569.3 (523.7–722)	0.03 *
SVRi (Din.s/cm⁵.m^2^)	1338 (1219–1603)	1046 (938.6–1184)	0.0007 *
EDV (mL)	150.5 (116.7–182.1)	117.1 (95.57–157.8)	0.1
EF (%)	57.46 (52.46–64.55)	74.50 (67.43–80.22)	0.0003 *

Data presented as median and interquartile range, * differences between groups *p* < 0.05, one-minute sit-to-stand test (1-STS), peripheral oxygen saturation (SpO_2_, %), heart rate (HR), stroke volume (SV), stroke volume index (SVi) cardiac output (CO), cardiac index (CI), Contractility Index (CTI), Ventricular Ejection Time (VET, ms), Systemic Vascular Resistance (SVR), Systemic Vascular Resistance Index (SVRi), End Diastolic Volume (EDV), Ejection Fraction (EF), meters for seconds (ms), liters per minute (L/min), beats per minute (bpm), percentage (%), milliliters per minute (mL/min).

**Table 3:** 1-STS results at rest, start, end, and recuperation moments.

**Stroke Volume**					
SV (mL)	COVID-19 (n = 36)	*p intragroup*	CONTROL (n = 11)	*p intragroup*	*p intergroup*
Rest	76.75 (62.68–84.85)	<0.0001	62.6 (57.2–85.2)	<0.0001	0.33
Start	71.05 (58.55–82.9)		77.3 (66.3–85.8)		0.53
End	89.8 (70.25–99.13)		89.3 (77.3–116.3)		0.79
Recuperation	79.1 (65.48–90.85)		77.3 (70.5–92.3)		0.9
**Stroke Volume Index**					
SVi (mL/m^2^)	COVID-19 (n = 36)	*p intragroup*	CONTROL (n = 11)	*p intragroup*	*p intergroup*
Rest	39.65 (34.53–44.93)	<0.0001	42.2 (33.9–46.6)	<0.0001	0.74
Start	36.35 (33.48–42.5)		43.8 (38–50.2)		0.03 *
End	44.35 (40.55–52.3)		54.4 (45.1–56.2)		0.11
Recuperation	40.45 (36.45–44.58)		45.8 (40.1–52.1)		0.05
**Heart Rate**					
HR (bpm)	COVID-19 (n = 36)	*p intragroup*	CONTROL (n = 11)	*p intragroup*	*p intergroup*
Rest	74 (64.25–81.75)	<0.0001	78 (67–83)	<0.0001	0.34
Start	88 (82–99.5)		102 (91–116)		0.01 *
End	129.5 (115–146)		133 (125–155)		0.26
Recuperation	88 (77.25–100)		89 (75–120)		0.66
**Cardiac Output**					
CO (L/min)	COVID-19 (n = 36)	*p intragroup*	CONTROL (n = 11)	*p intragroup*	*p intergroup*
Rest	5.4 (4.42–6.27)	<0.0001	5.2 (3.9–6.3)	<0.0001	0.89
Start	6.35 (5.05–7.97)		7 (6.5–9.3)		0.09
End	11.05 (8.8–13.18)		11.9 (9.7–15.6)		0.39
Recuperation	6.75 (5.3–8.5)		7.7 (6.2–8.8)		0.49
**Cardiac Index**					
CI (mL/min/m^2^)	COVID-19 (n = 36)	*p intragroup*	CONTROL (n = 11)	*p intragroup*	*p intergroup*
Rest	2.65 (2.5–3.3)	<0.0001	3.2 (2.6–3.6)	<0.0001	0.16
Start	3.3 (2.9–4.17)		4.4 (4–5.3)		0.002 *
End	5.9 (4.82–6.47)		7 (6.5–7.5)		0.02 *
Recuperation	3.7 (2.8–4.2)		4.2 (3.5–5)		0.07
**Contractility Index**					
CTI	COVID-19 (n = 36)	*p intragroup*	CONTROL (n = 11)	*p intragroup*	*p intergroup*
Rest	136.7 (88.15–164.9)	<0.0001	218 (187.1–298.5)	0.0003	0.0003 *
Start	131 (95,05–190,7)		319.2 (260.8–349.8)		<0.0001 *
End	225.4 (142.5–304.8)		350 (277.6–653.1)		0.002 *
Recuperation	201.2 (166–290.7)		431.1 (304.7–516.5)		0.0005 *
**Ventricular Ejection Time**					
VET (ms)	COVID-19 (n = 36)	*p intragroup*	CONTROL (n = 11)	*p intragroup*	*p intergroup*
Rest	314 (267–399.5)	<0.0001	272 (236–320)	0.0534	0.25
Start	292 (225–334)		272 (236–368)		0.79
End	234 (204–282.7)		252 (196–304)		0.53
Recuperation	338.4 (296–408)		372 (296–400)		0.77
**Systemic Vascular Resistance Index**					
SVRi (Din.s/cm⁵.m^2^)	COVID-19 (n = 36)	*p intragroup*	CONTROL (n = 11)	*p intragroup*	*p intergroup*
Rest	2366 (2093–2853)	<0.0001	2079 (1756–2382)	<0.0001	0.09
Start	2031 (1639–2323)		1432 (1337–1549)		0.001 *
End	1160 (1040–1376)		962 (780–1105)		0.01 *
Recuperation	1853 (1631–2222)		1623 (1272–1949)		0.05
**Systemic Vascular Resistance**					
SVR (Din.s/cm⁵)	COVID-19 (n = 36)	*p intragroup*	CONTROL (n = 11)	*p intragroup*	*p intergroup*
Rest	1287 (1121–1448)	<0.0001	1174 (961–1536)	<0.0001	0.01 *
Start	1038 (898.5–1239)		886 (668–971)		0.88
End	622 (533–734.5)		510 (461–710)		0.2
Recuperation	1009 (852.5–1170)		913 (735–1080)		0.31
**End Diastolic Volume**					
EDV (mL)	COVID-19 (n = 36)	*p intragroup*	CONTROL (n = 11)	*p intragroup*	*p intergroup*
Rest	138.3 (113.2–172.9)	0.0162	96.3 (85.8–143.5)	0.0598	0.02 *
Start	145.2 (106.9–175.8)		97.8 (89.7–136.1)		0.02 *
End	147.8 (113.6–180.3)		111.4 (86.5–143.4)		0.04 *
Recuperation	135.4 (111.2–150.6)		103.7 (82.5–141.1)		0.03 *
**Ejection Fraction**					
EF (%)	COVID-19 (n = 36)	*p intragroup*	CONTROL (n = 11)	*p intragroup*	*p intergroup*
Rest	52.85 (44.95–59.5)	<0.0001	66.7 (60.4–73.8)	0.0038	0.0004 *
Start	53.7 (45.18–64)		71.2 (69.1–75)		<0.0001 *
End	60.7 (54.3–68.48)		77.2 (68.6–89.5)		0.0005 *
Recuperation	60.5 (53.8–66.98)		76.7 (66.9–87)		0.0006 *

Data presented as median and interquartile range, * differences between groups *p* < 0.05, heart rate (HR), stroke volume (SV), stroke volume index (SVi) cardiac output (CO), cardiac index (CI), Contractility Index (CTI), Ventricular Ejection Time (VET), Systemic Vascular Resistance (SVR), Systemic Vascular Resistance Index (SVRi, ^2^), End Diastolic Volume (EDV), Ejection Fraction (EF), meters for seconds (ms), liters per minute (L/min), beats per minute (bpm), percentage (%), milliliters per minute (mL/min).

**Table 4:** 6MWT results and cardiorespiratory parameters.

6MWT	COVID-19 (n = 36)	CONTROL (n = 11)	*p*
6MWT (meters)	524.3 (478.5–579)	630 (610–660)	0.0001 *
6MWT (% pred)	91.44 (81.64–98.84)	107.9 (98.14–114.4)	<0.0001 *
SpO_2_ post (%)	98 (97–98)	98 (96–98)	0.79
HR post (bpm)	106.5 (96.25–120.8)	120(107–142)	0.07
Dyspnea post (Borg 0–10)	3 (2–4)	3(2–5)	0.43
Fatigue post (Borg 0–10)	3.5 (2–5.75)	5(3–7)	0.35

Data presented as median and interquartile range, * differences between groups *p* < 0.05, six-minute walk test (6MWT), peripheral oxygen saturation (SpO_2_, %), heart rate (HR), beats per minute (bpm), percentage (%), percentage of predicted (% of pred).

## Data Availability

Data are available on request from the authors. The data that support the findings of this study are available from the corresponding author (VR) upon reasonable request.
